# Duplex Analysis of Cannulated Vessels in Peripheral Veno-Arterial Extracorporeal Membrane Oxygenation

**DOI:** 10.3390/medicina58050671

**Published:** 2022-05-18

**Authors:** Jorik Simons, Sandra Agricola, Jeroen Smets, Renske Metz, Silvia Mariani, Marie-José Vleugels, Reinier R. Smeets, Walther N. K. A. van Mook, Barend Mees, Roberto Lorusso

**Affiliations:** 1 Cardio-Thoracic Surgery, Maastricht University Medical Center, P. Debyelaan 25, 6229 HX Maastricht, The Netherlands; s.mariani1985@gmail.com (S.M.); mjpj.vleugels@mumc.nl (M.-J.V.); roberto.lorussobs@gmail.com (R.L.); 2 Intensive Care Medicine, Maastricht University Medical Center, P. Debyelaan 25, 6229 HX Maastricht, The Netherlands; w.van.mook@mumc.nl; 3 Cardiovascular Research Institute Maastricht, Maastricht University, Universiteitssingel 50, 6229 ER Maastricht, The Netherlands; rr.smeets@gmail.com (R.R.S.); barend.mees@mumc.nl (B.M.); 4 Vascular Surgery, Maastricht University Medical Center, P. Debyelaan 25, 6229 HX Maastricht, The Netherlands; sandra.agricola@mumc.nl (S.A.); jeroen.smets@mumc.nl (J.S.); renske.metz@mumc.nl (R.M.); 5 School of Health Professions Education, Maastricht University, Universiteitssingel 60, 6229 ER Maastricht, The Netherlands; 6 Academy for Postgraduate Medical Training, Maastricht University Medical Center, P. Debyelaan 25, 6229 HX Maastricht, The Netherlands

**Keywords:** ECMO 1, veno-arterial 2, V-A 3, femoral 4, duplex 5, ultrasound 6

## Abstract

*Background and objectives:* Veno-arterial extracorporeal membrane oxygenation (V-A ECMO) cannulas have major repercussions on vascular hemodynamics that can potentially lead to limb ischemia. Duplex ultrasound enables the non-invasive analysis of vascular hemodynamics. This study aims to describe the duplex parameters of the femoral vessels during V-A ECMO support, investigate differences between cannulated and non-cannulated vessels, and analyze the variations in the case of limb ischemia and intra-aortic balloon pumps (IABPs). *Methods:* Nineteen adults (≥18 years), supported with femoro-femoral V-A ECMO, underwent a duplex analysis of the superficial femoral arteries (SFAs) and veins (FVs). Measured parameters included flow velocities, waveforms, and vessel diameters. *Results:* 89% of patients had a distal perfusion cannula during duplex analysis and 21% of patients developed limb ischemia. The mean peak systolic flow velocity (PSV) and end-diastolic flow velocity (EDV) of the SFAs on the cannulated side were, respectively, 42.4 and 21.4 cm/s. The SFAs on the non-cannulated side showed a mean PSV and EDV of 87.4 and 19.6 cm/s. All SFAs on the cannulated side had monophasic waveforms, whereas 63% of the SFAs on the non-cannulated side had a multiphasic waveform. Continuous/decreased waveforms were seen in 79% of the FVs on the cannulated side and 61% of the waveforms of the contralateral veins were respirophasic. The mean diameter of the FVs on the cannulated side, in patients who developed limb ischemia, was larger compared to the FVs on the non-cannulated side with a ratio of 1.41 ± 0.12. The group without limb ischemia had a smaller ratio of 1.03 ± 0.25. *Conclusions:* Femoral cannulas influence flow velocities in the cannulated vessels during V-A ECMO and major waveforms alternations can be seen in all SFAs on the cannulated side and most FVs on the cannulated side. Our data suggest possible venous stasis in the FV on the cannulated side, especially in patients suffering from limb ischemia.

## 1. Introduction

Extracorporeal membrane oxygenation (ECMO) is an increasingly used life-saving support measure for patients with severe refractory cardio-respiratory failure [1,2]. Since ECMO utilizes cannulas to extract and return multiple liters of blood per minute, it has a substantial influence on the patient’s hemodynamics. This is more pronounced in veno-arterial (V-A) than in veno-venous (V-V) ECMO since V-A is mostly used in cardiac failure and, thus, as circulatory support.

Several vessels can be used for cannulation, with the common femoral artery and vein being predominantly used in peripheral V-A ECMO [3]. Arterial and venous cannulas have an outer diameter of around 19–23 French and 25–27 French, respectively. Cannula diameter is selected to correlate with the patient’s body size. However, due to their relatively large size, the intravascular portion of these cannulas has significant repercussions on the hemodynamics of the cannulated vessels. For example, limb ischemia due to arterial hypoperfusion is seen in 10–30% of peripheral V-A ECMOs, and a distal arterial perfusion cannula (DPC) is recommended to guarantee adequate blood flow to the limb [4,5,6,7,8,9]. Besides arterial-related hypoperfusion, vein cannulation may limit the venous blood drainage from the cannulated limb, thereby generating venous blood stasis, which may, independently or concomitantly with arterial hypoperfusion, predispose to limb ischemia [10]. Based on the mentioned factors, bilateral cannulation—performing venous cannulation in one leg and arterial cannulation in the other—is thought to be a protective measure for limb ischemia due to a more optimized interplay between arterial hypoperfusion and venous stasis [7].

Due to the rather high incidence of cannulated limb ischemia, perfusion monitoring represents a critical aspect of complication prevention and assessment. Based on its non-invasive nature and bedside availability, duplex analysis can be used to measure flow velocities, waveforms, and vessel diameters to estimate limb perfusion. Due to its non-invasive nature and bedside availability, duplex analysis seems to be ideally suited to examine the status of limb perfusion in ECMO patients.

Despite the relevance of the clinical problem and the large diffusion of duplex analysis in intensive care units, limited literature is available regarding the hemodynamic repercussion of cannulas in the cannulated vessels and no descriptive research has been performed concerning arterial and venous duplex parameters in cannulated and non-cannulated vessels.

Therefore, this observational study was designed to assess the duplex parameters of the superficial femoral arteries (SFAs) and femoral veins (FVs) of the cannulated and non-cannulated limbs in ECMO patients. We aimed to determine reference duplex values, investigate differences between cannulated and non-cannulated vessels, and analyze the variations in the case of limb ischemia and the use of intra-aortic balloon pumps (IABPs).

## 2. Materials and Methods

This observational study prospectively included consecutive patients supported with V-A ECMO from 2019 through 2021 in a university medical center in the Netherlands. Inclusion criteria were: (1) age ≥ 18 years; (2) treatment with V-A, venoveno-arterial (VV-A), or veno-arterovenous (V-AV) ECMO; (3) femoral vessel cannulation; and (4) the availability of a vascular ultrasound technician. This study received approval from the local ethical committee (2021-2996).

After inclusion, a duplex ultrasound was performed as early as possible (usually within 24 h from ECMO initiation) during the ECMO treatment. Further post-analysis was performed according to the consensus statement from the Society for Vascular Medicine and Society for Vascular Ultrasound [11].

Demographic data, baseline characteristics, ECMO and cannula characteristics, duplex parameters, and outcomes were collected and analyzed. DPC flow was measured via a flow sensor on the circuit tubing. In the case of the use of a cannula with an integrated DPC, it was not possible to measure this parameter. Duplex measurements (flow velocities, vessel diameter, and waveform patterns) were considered primary outcome measures. Moreover, in order to preliminarily compare the outcome measures from the cannulated side with the non-cannulated side, ratios were calculated by dividing the outcome measure of the cannulated side by the non-cannulated side. A descriptive sub-analysis of the primary outcome measures was performed in patients with or without a clinical diagnosis of limb ischemia and patients with and without IABP (which was used for left ventricular unloading). Secondary outcomes were limb-related complications (bleeding at cannulation site, compartment syndrome/fasciotomy, thrombectomy, and arterial vessel repair), and mortality during intensive care unit stay.

Continuous variables were reported as mean and standard deviation (±SD) and categorical variables were reported as counts and percentages. Based on the explorative, descriptive, and feasibility nature of this study, no formal sample size calculation was performed. Consequently, no statistical inference was conducted.

## 3. Results

### 3.1. Baseline Characteristics

The cohort consisted of nineteen patients who were predominantly males (*n* = 11, 57.9%), with a mean age of 56 (range: 24–72) years, and a body mass index of 27 (range: 18–40) kg/m^2^ (Table 1). Mono-organ failure was present in 78.9% of patients and post-cardiotomy cardiac failure was the most frequent indication for ECMO. Sixteen patients were treated with V-A ECMO (84.2%), two patients with V-AV (10.5%), and one patient with VV-A ECMO (5.3%; Table 2) [12]. During duplex analysis, 31.6% of the cohort was additionally treated with an IABP positioned in the contralateral common femoral artery.

At the initiation of ECMO treatment, norepinephrine at a rate of 0.25–0.5 mcg/kg/min was necessary for 42.1% of the cohort. Additionally, the mean ECMO blood flow was 3.6 L/minute, the mean airflow was 2.5 L/minute, and the mean fraction inspired oxygen was 57%. Most patients underwent unilateral femoral cannulation with 19 Fr arterial and 25 Fr venous cannulas (Table 3). Seventeen patients (89%) received a prophylactic DPC. Left and right limb near-infrared spectroscopy (NIRS) values at ECMO initiation were 64 ± 18% on the side with the arterial cannula and 59 ± 21% on the side without the arterial cannula.

### 3.2. Duplex Analysis of Superficial Femoral Arteries

**Figure 1:**
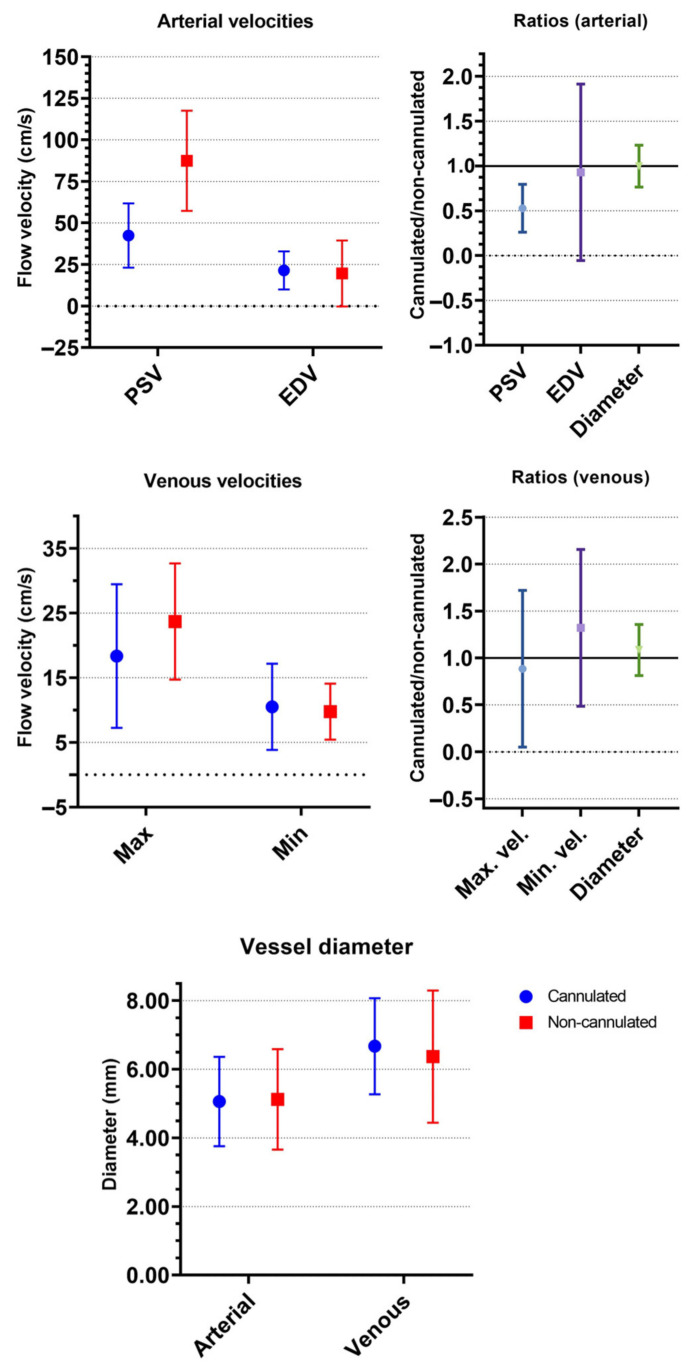
This figure shows the mean arterial peak systolic velocity (PSV) and end-diastolic velocity (EDV) for both the cannulated and non-cannulated arteries. Mean maximum (max) and minimum (min) velocities, including velocity difference, are also shown for the femoral veins. Mean ratios and vessel diameters are also reported. All graphs show standard deviations.

Table 4 shows the results of the duplex analysis of the SFAs on the cannulated and non-cannulated sides. The mean PSV of the SFAs on the cannulated and non-cannulated sides were 42.4 ± 19.4 and 87.4 ± 30.1 cm/s, respectively, resulting in a mean ratio (PSV of the cannulated side divided by the PSV of the non-cannulated side) of 0.53 ± 0.27. The mean EDV was, respectively, 21.4 ± 11.4 and 19.6 ± 19.8 cm/s, with a mean ratio of 0.93 ± 0.98. The SFA diameter ratio was 1.00 ± 0.23. Figure 1 shows the flow velocities, ratios, and diameters of the superficial femoral arteries and veins.

All SFAs on the cannulated side showed monophasic flow patterns—52.6% showed minimal phasic, monophasic flow patterns, while 21.1% showed low resistive flow patterns. No high resistive patterns were seen. The majority of SFAs on the non-cannulated side had a multiphasic and/or IABP-related flow pattern (63.2%). High, intermediate, and low resistive monophasic flow patterns were observed in, respectively, 10.5%, 21.1%, and 5.3% of patients. No low resistive or minimal phasic flow patterns were observed in the SFAs of the non-cannulated side.

Mean ECMO flow during duplex analysis was 3.09 ± 0.94 L/minute. DPC flow was measured at 261.9 ± 55.21 mL/minute.

### 3.3. Duplex Analysis of Femoral Veins

The mean maximum velocities of the VFs on the cannulated and non-cannulated sides were 18.4 ± 11.1 and 23.7 ± 9 cm/s, respectively. The mean ratio between these parameters was 0.89 ± 0.83. The mean minimum velocity of both veins was: 10.5 ± 6.7 cm/s for the cannulated side and 9.8 ± 4.3 cm/s for the non-cannulated side. Flow pattern analysis showed a shift toward flow patterns that were respirophasic or respirophasic with a cardiac cycle in the FVs on the non-cannulated side whereas the FVs on the cannulated side had a continuous flow pattern in 47.4% of patients (Table 5).

### 3.4. Sub-Analysis of Limb Ischemia

A sub-analysis of the SFAs of patients who developed limb ischemia showed a mean PSV and EDV ratio of 0.69 ± 0.48 and 1.68 ± 1.38, respectively. Furthermore, patients who developed limb ischemia showed monophasic flow patterns in all SFAs on the cannulated side and multiphasic flow in half of the SFAs on the non-cannulated side.

A duplex analysis of FVs in patients who developed limb ischemia showed higher mean maximum and minimum flow velocities. The mean diameter of the FVs on the cannulated side, in the patients who developed limb ischemia, was larger compared to the FVs on the non-cannulated side with a ratio of 1.41 ± 0.12. The group without limb ischemia had a smaller ratio of 1.03 ± 0.25. Notably, in only two of the four patients who developed limb ischemia, it was possible to determine vessel diameters.

### 3.5. Sub-Analysis of IABP

An analysis of arterial values measured in patients with and without IABP showed lower mean EDV for both the SFAs on the cannulated and non-cannulated sides in the group with IABP compared to the group without IABP (Table 6). Vessel diameter was similar between both cohorts and flow patterns were highly different due to the in- and deflation of the balloon.

In the sub-analysis of patients with and without IABP, venous duplex parameters were predominantly comparable. Notably, there was a relatively low maximum venous flow in the FVs on the cannulated side of patients with IABP (Table 7).

### 3.6. Outcomes

Outcomes are reported in Table 8. Overall, 21% of patients developed limb ischemia and 5.3% of patients developed a compartment syndrome/required a fasciotomy during ECMO, 15.8% underwent thrombectomy during ECMO, and 5.3% of patients required arterial vessel repair during ECMO. ICU mortality was 47.4%.

## 4. Discussion

### 4.1. Main Findings

To our knowledge, this is the first study investigating duplex ultrasound parameters of the SFAs and FVs in V-A ECMO patients with peripheral femoral cannulation. In this prospective cohort, we observed:

- Normal PSV with, in most patients, multiphasic flow patterns in the SFAs on the non-cannulated side.
- Respirophasic (with or without cardiac cycles) flow patterns in the FVs on the non-cannulated side.
- Decreased PSV and monophasic flow patterns in the SFAs on the cannulated side.
- Non-phasic flow patterns in the majority of the FVs on the cannulated side.
- Suggestions that continuous flow, with a small difference between the maximum and minimum flow velocity in the FVs on the cannulated side and a large vein diameter ratio, indicate venous stasis, which might be a risk factor for limb ischemia.

### 4.2. Arterial and Venous Duplex Parameters

Femoral cannulas have a significant influence on the vascular hemodynamics of the limb and they play a pivotal role in the development of limb ischemia in patients supported with peripheral ECMO [7]. Advanced and non-invasive monitoring techniques, for example, vascular duplex ultrasound, might be a helpful tool to monitor the perfusion status of the cannulated and non-cannulated limb, helping with the clinical workout of limb ischemia during ECMO.

The arterial and venous cannulas have major repercussions on the vascular hemodynamics of the limb by arterial obstruction and venous congestion [4,5,6,7,8,9,10]. On one side, DPCs play a major role in the continuous supply of oxygenated blood to the distal limb [13]. On the other side, variations in native cardiac output, ECMO flow, IABP support, and vasoactive drugs further impact limb hemodynamics. 

Stiegler et al., reported normal values for the PSV in the SFA of 73 to 90 cm/s [14]. This is in line with the mean PSV measured in the SFAs on the non-cannulated side. However, the mean PSV of the SFAs on the cannulated side was lower compared to the aforementioned reference values. This is most likely due to common femoral artery and iliac obstruction by the arterial cannula [15]. The SFA flow pattern analysis is supportive of this observation.

Multiphasic flow patterns are considered normal in the femoral arteries of a healthy population and monophasic flow patterns (i.e., tardus-parvus waveform) indicate vascular pathology, most likely due to proximal stenotic pressure loss [14,15,16]. We observed that all SFAs on the cannulated side had a monophasic flow pattern, due to the presence of the arterial cannula in the proximal part of the SFA which replicates the effects of proximal stenosis. In the SFAs on the non-cannulated side, most patients showed multiphasic flow patterns indicating the absence of severe proximal stenosis and an efficient transmission of the pulse pressure on the non-cannulated side. Notably, when the flow pattern was described as monophasic, it was characterized by a higher resistive flow pattern compared to the SFAs on the cannulated side. High resistive flow patterns indicate a high pulse pressure traveling down the arterial tree and result in a minimal flow during diastole. 

Smet et al., reported a direct relationship between increasing EDVs and the grade of aortoiliac stenosis in patients with peripheral artery occlusive disease [17]. We observed high mean EDVs in both the SFAs on the cannulated and non-cannulated sides. The high mean EDV in the SFAs on the cannulated side could be explained by the DPC generating a constant flow towards the limb and by the obstruction induced by the cannula. However, the high mean EDV on the non-cannulated side could be the result of constant ECMO flow since, as noted earlier, the mean PSV and flow patterns make stenosis less probable. An IABP in situ could potentially be an obstructive component; however, due to its relatively small sheath size (7.5–8 Fr), this effect is likely to be limited.

The slightly lower ECMO blood flow during duplex analysis, compared to the blood flow at initiation, can be explained by the initial stabilization of the patient before the duplex analysis (most duplex measurements were made within 24 h after ECMO initiation).

In the femoral veins of healthy people, normal waveforms are observed with both respiratory and cardiac cycles [18,19,20]. During spontaneous inspiration, a reduction in right atrial pressure and an increase in venous return results in a respirophasic flow pattern. In intubated patients, positive-pressure ventilation results in increased right atrial and abdominal pressure and, thus, the damping of the signal [18]. Flow analysis in the cohort herein described showed a shift towards respirophasic (with or without cardiac cycles) flow patterns for the FVs of the non-cannulated side. This was also reflected by the difference between the maximum and minimum velocity: a relatively large difference between the maximum and minimum velocity was observed in the FVs on the non-cannulated side. However, FVs on the cannulated side showed a more damped or continuous flow pattern, indicating a decreased effect of the changing venous return. This could be explained by the obstructive nature of the venous cannula.

### 4.3. Duplex Parameters in Limb Ischemia and IABP Support

The mean PSV of the SFAs on the non-cannulated side was similar between patients who experienced limb ischemia and patients with no ischemic problems. However, mean EDV showed higher values in patients with limb ischemia (32.5 ± 22.3 vs. 16.2 ± 18.4 cm/s). This might be due to the influence of IABP flow in patients who did not develop limb ischemia. Sub-analyzing the cohort with IABP in situ namely shows a relatively low mean EDV, which could decrease the mean EDV in the no-limb ischemia group since no IABP signals were registered in the ischemia group. Another reason could be the position of the measurement location (relatively close to the cannula), resulting in high post-stenotic flow velocities. A comparison of more distal SFA flow velocities could gather further insight into this possible explanation. Flow pattern analysis does not seem to be a factor differentiating limb ischemia in this cohort.

On the venous side, the difference between the maximum and minimum velocity was smaller for the limb ischemia cohort compared to the cohort without limb ischemia. The flow pattern analysis showed a shift towards continuous venous flow in the FVs of patients who developed limb ischemia, whereas the variation of flow patterns in patients who did not develop limb ischemia was broader. This could indicate that patients with continuous flow and a small difference between the maximum and minimum velocity in the cannulated vein could be more at risk for the development of limb ischemia.

Additionally, the FVs on the cannulated side in the limb ischemia cohort were 1.41 ± 0.12 times larger compared to the non-cannulated side, whereas the sizes in the group, who did not develop limb ischemia were near equal (ratio cannulated/non-cannulated: 1.03 ± 0.25). This could suggest a higher venous pressure in patients who developed limb ischemia. The reason for such a higher pressure might be the stasis generated by increased resistances or vein occlusion induced by the venous cannula. Since veins have relatively little elastic tissue and muscle, they have a typical high vascular compliance, which could explain the relatively comparable femoral vein size and the absence of venous stasis in the group without limb ischemia [21].

### 4.4. Limitations and Strengths of the Study

The cohort was designed as a pilot study and limited by a relatively low number of patients. Moreover, a limited number of cases available for sub-analysis was a major limitation. A larger cohort is needed to perform further comparative statistical analyses. Furthermore, DPC was used in most patients and it might have significantly influenced the duplex measurements. However, the placement of a DPC at the time of ECMO initiation is nowadays common practice in many centers. For this reason, our results could be transferable to most current ECMO patients. A proportion of our cohort had an IABP, thus altering the flow patterns of both the cannulated and non-cannulated vessels. This bias was addressed through sub-group analysis. 

The prospective character of this study made it less susceptible to selection bias. Additionally, as few as possible exclusion criteria were used. Duplex measurements were made by an experienced ultrasound technician who specialized in vascular ultrasound, which increased the reliability and validity of the measurements.

## 5. Conclusions

Femoral cannulas have a significant influence on flow velocities and patterns in the cannulated vessels during V-A ECMO. Major alternations in waveforms were seen in all SFAs on the cannulated side and in most FVs on the cannulated side. These results suggest that continuous flow, with a small difference between the maximum and minimum flow velocity in the FV on the cannulated side, and a large vein diameter ratio, might indicate venous stasis and, thus, the risk of limb ischemia.

## Figures and Tables

**Table 1:** Baseline patients’ characteristics.

	Overall Population
	*N* = 19
Gender	
Male	11 (57.9%)
Female	8 (42.1%)
Age (years)	56 ± 14
Body Mass Index (kg/m²)	27.1 ± 5.4
Body Surface Area (m²)	1.95 ± 0.16
Type of organ failure	
Cardiac	15 (78.9%)
Cardiac and kidney	3 (15.8%)
Cardiac and pulmonary	1 (5.3%)
History	
Hypertension	7 (36.8%)
Myocardial infarction at presentation	6 (31.6%)
Endocarditis	5 (26.3%)
Recent atrial fibrillation	5 (26.3%)
Hypercholesterolemia	5 (26.3%)
Asthma	3 (15.8%)
Diabetes	3 (15.8%)
Myocardial infarction (<90 days before presentation)	2 (10.5%)
Cardiothoracic surgery (<1 year before presentation)	2 (10.5%)
Recent acute kidney injury (<90 days before presentation)	2 (10.5%)
Peripheral artery disease	1 (5.3%)
Deep venous thrombosis	1 (5.3%)
Chronic obstructive pulmonary disease	1 (5.3%)
Cardiothoracic surgery (<90 days before presentation)	1 (5.3%)

**Table 2:** Hemodynamic, ventilatory, and extracorporeal membrane oxygenation characteristics at initiation. Values are reported as count and percentage or mean and standard deviation. ECPR: extracorporeal cardiopulmonary resuscitation, ECMO: extracorporeal membrane oxygenation, IABP: intra-aortic balloon pump, V-A: veno-arterial, V-AV: veno-arterovenous, VV-A venoveno-arterial.

	Overall Population *N* = 19
Indication	
Postcardiotomy, ventricular failure	12 (63.2%)
Cardiogenic shock, right ventricular failure	2 (10.5%)
ECPR, right ventricular failure	2 (10.5%)
Post-myocardial infarction, ventricular septum rupture	1 (5.3%)
Post-myocardial infarction	1 (5.3%)
Respiratory failure, pulmonary hypertension	1 (5.3%)
Mode	
V-A	16 (84.2%)
V-AV	2 (10.5%)
VV-A	1 (5.3%)
Mean ECMO settings (at initiation)	
Blood flow (L/min)	3.6 ± 0.6
Air flow (L/min)	2.5 ± 1.1
Fraction inspired oxygen (%)	57 ± 22
pH	7.30 ± 0.17
pCO_2_ (mmHg)	4.6 ± 1.3
pO_2_ (mmHg)	18 ± 7.2
HCO_3_^−^ (mmol/L)	16.6 ± 5.5
Norepinephrine (mcg/kg/min)	
None	1 (5.0%)
0–0.2	4 (21.1%)
0.25–0.5	8 (42.1%)
>0.5	6 (31.6%)
Hemodynamic support agents (mean, count)	1.6 ± 0.8
IABP (during duplex)	
No	13 (68.4%)
Yes	6 (31.6%)

**Table 3:** Cannula-related characteristics. DPC: distal perfusion cannula, Fr: French, NIRS: near-infrared spectroscopy.

	Overall Population, *N* = 19	
	Arterial Cannula	Venous Cannula
Cannulation Mode		
Unilateral	16 (84%)	
Bilateral	3 (16%)	
Cannula size		
15 Fr	2 (11%)	
19 Fr	12 (63%)	
21 Fr	5 (26%)	2 (11%)
23 Fr		2 (11%)
25 Fr		15 (79%)
DPC (at time of duplex)		
No	1 (5%)	
Yes	17 (89%)	
After	1 (5%)	
Limb NIRS at initiation of ECMO		
Side with arterial cannula	64 ± 18%	
Side without non-arterial cannula	59 ± 21%	

**Table 4:** Duplex measurements of the superficial femoral artery, reporting the total cohort, and patients with and without limb ischemia. Measurements of the cannulated and non-cannulated side are displayed. The ratios (cannulated versus non-cannulated) are based on the mean of the patient’s ratios. Flow pattern description was based on the consensus statement from the Society for Vascular Medicine (SVM) and the Society for Vascular Ultrasound (SVU) [11]. DPC: distal perfusion cannula, EDV: end-diastolic flow velocity, IABP: intra-aortic balloon pump, PSV: peak-systolic flow velocity.

Superficial Femoral Artery	Overall Population			No Limb Ischemia			Limb Ischemia		
	Cannulated	Non-Cannulated	Ratio	Cannulated	Non-Cannulated	Ratio	Cannulated	Non-Cannulated	Ratio
	*N* = 19	*N* = 19		*N* = 15	*N* = 15		*N* = 4	*N* = 4	
PSV, mean (cm/s)	42.4 ± 19.4	87.4 ± 30.1	0.53 ± 0.27	39.7 ± 15.7	87.3 ± 31.8	0.49 ± 0.19	52.7 ± 30.4	87.8 ± 27	0.69 ± 0.48
EDV, mean (cm/s)	21.4 ± 11.4	19.6 ± 19.8	0.93 ± 0.98	18.6 ± 9	16.2 ± 18.4	0.73 ± 0.8	32.1 ± 14.5	32.5 ± 22.3	1.68 ± 1.38
Diameter, mean (mm)	5.1 ± 1.3	5.1 ± 1.5	1.00 ± 0.23	5.1 ± 1.4	5.2 ± 1.5	0.98 ± 0.23	5.1 ± 0.9	4.6	1.24
Flow pattern									
Multiphasic	0 (0%)	8 (42.1%)		0 (0%)	6 (40%)		0 (0%)	2 (50%)	
IABP	0 (0%)	4 (21.1%)		0 (0%)	4 (26.7%)		0 (0%)	0 (0%)	
Monophasic									
High resistive	0 (0%)	2 (10.5%)		0 (0%)	1 (6.7%)		0 (0%)	1 (25%)	
Intermediate resistive	2 (10.5%)	4 (21.1%)		2 (13.3%)	3 (20%)		0 (0%)	1 (25%)	
Low resistive	4 (21.1%)	1 (5.3%)		3 (20%)	1 (6.7%)		1 (25%)	0 (0%)	
Minimal phasic	10 (52.6%)	0 (0%)		7 (46.7%)	0 (0%)		3 (75%)	0 (0%)	
Plus IABP signal	3 (15.8%)	0 (0%)		3 (20%)	0 (0%)		0 (0%)	0 (0%)	
Blood flow during duplex, mean (L/min)	3.09 ± 0.94			3.15 ± 0.96			2.85 ± 0.9		
DPC									
Flow (mL/min)	261.9 ± 55.21			253.63 ± 59.34			295 ± 7.07		
Placement									
Direct	17 (89.5%)			15 (100%)			2 (50%)		
Delayed	1 (5.3%)			0 (0%)			1 (25%)		
None	1 (5.3%)			0 (0%)			1 (25%)		

**Table 5:** Duplex measurements of the femoral vein, reporting the total cohort, and patients with and without limb ischemia. Measurements of the cannulated and non-cannulated side are displayed. The ratios (cannulated versus non-cannulated) are based on the mean of the patient’s ratios. Flow pattern description was based on the consensus statement from the Society for Vascular Medicine (SVM) and the Society for Vascular Ultrasound (SVU) [11].

Femoral Vein	Overall Population			No limb Ischemia			Limb Ischemia		
	Cannulated	Non-Cannulated	Ratio	Cannulated	Non-Cannulated	Ratio	Cannulated	Non-Cannulated	Ratio
	*N* = 19	*N* = 18		*N* = 15	*N* = 14		*N* = 4	*N* = 4	
Maximum velocity, mean (cm/s)	18.4 ± 11.1	23.7 ± 9	0.89 ± 0.83	17.9 ± 11.6	22.6 ± 7.2	0.92 ± 0.93	20.3 ± 10.3	27.5 ± 14.4	0.77 ± 0.42
Minimum velocity, mean (cm/s)	10.5 ± 6.7	9.8 ± 4.3	1.32 ± 0.84	9.6 ± 6.2	9.1 ± 4.2	1.3 ± 0.73	14 ± 8.2	12.2 ± 4.4	1.39 ± 1.29
Diameter, mean (mm)	6.7 ± 1.4	6.4 ± 1.9	1.08 ± 0.27	6.7 ± 1.5	6.6 ± 2	1.03 ± 0.25	6.8 ± 1 (n = 2)	4.8 ± 0.3 (n = 2)	1.41 ± 0.12
Flow pattern									
Respirophasic and cardiac cycle	1 (5.3%)	5 (27.8%)		1 (6.7%)	4 (28.6%)		0 (0%)	1 (25%)	
Respirophasic	3 (15.8%)	6 (33.3%)		3 (20%)	4 (28.6%)		0 (0%)	2 (50%)	
Decreased	3 (15.8%)	1 (5.6%)		3 (20%)	1 (7.1%)		0 (0%)	0 (0%)	
Pulsatile (cardiac cycle)	3 (15.8%)	4 (22.2%)		2 (13.3%)	3 (21.4%)		1 (25%)	1 (25%)	
Continuous	9 (47.4%)	2 (11.1%)		6 (40%)	2 (14.3%)		3 (75%)	0 (0%)	

**Table 6:** Duplex measurements of the superficial femoral artery, reporting patients with and without IABP. Measurements of the cannulated and non-cannulated sides are displayed. The ratios (cannulated versus non-cannulated) are based on the mean of the patient’s ratios. Flow pattern description was based on the consensus statement from the Society for Vascular Medicine (SVM) and the Society for Vascular Ultrasound (SVU) [11]. DPC: distal perfusion cannula, EDV: end-diastolic flow velocity, IABP: intra-aortic balloon pump, PSV: peak-systolic flow velocity.

Superficial Femoral Artery	Without IABP			With IABP		
	Cannulated	Non-Cannulated	Ratio	Cannulated	Non-Cannulated	Ratio
	*N* = 13	*N* = 13		*N* = 6	*N* = 6	
PSV, mean (cm/s)	48.7 ± 18.8	89.2 ± 22.9	0.59 ± 0.29	28.8 ± 13.1	83.6 ± 44.5	0.39 ± 0.16
EDV, mean (cm/s)	25.3 ± 11.1	25 ± 14.7	1.31 ± 0.82	13 ± 7.1	8 ± 25.7	0.1 ± 0.82
Diameter, mean (mm)	5.0 ± 1.2	5.3 ± 1.5	0.94 ± 0.18	5.1 ± 1.6	4.8 ± 1.5	1.09 ± 0.29
Flow pattern						
Multiphasic	0 (0%)	7 (53.8%)		0 (0%)	1 (16.7%)	
IABP	0 (0%)	0 (0%)		0 (0%)	4 (66.7%)	
Monophasic						
High resistive	0 (0%)	2 (15.4%)		0 (0%)	0 (0%)	
Intermediate resistive	2 (15.4%)	4 (30.8%)		0 (0%)	0 (0%)	
Low resistive	4 (30.8%)	0 (0%)		0 (0%)	1 (16.7%)	
Minimal phasic	7 (53.8%)	0 (0%)		3 (50%)	0 (0%)	
Plus IABP signal	0 (0%)	0 (0%)		3 (50%)	0 (0%)	
Blood flow during duplex, mean (L/min)	3.10 ± 0.94			3.07 ± 1.01		
DPC						
Flow (mL/min)	287.5 ± 19.94			223.5 ± 72.15		
Placement						
Direct	11 (84.6%)			6 (100%)		
Delayed	1 (7.7%)			0 (0%)		
None	1 (7.7%)			0 (0%)		

**Table 7:** Duplex measurements of the femoral vein, reporting patients with and without IABP. Measurements of the cannulated and non-cannulated sides are displayed. The ratios (cannulated versus non-cannulated) are based on the mean of the patient’s ratios. Flow pattern description was based on the consensus statement from the Society for Vascular Medicine (SVM) and the Society for Vascular Ultrasound (SVU) [11]. IABP: intra-aortic balloon pump.

Femoral Vein	Without IABP			With IABP		
	Cannulated	Non-Cannulated	Ratio	Cannulated	Non-Cannulated	Ratio
	*N* = 13	*N* = 12		*N* = 6	*N* = 6	
Maximum velocity, mean (cm/s)	20.3 ± 13.1	23.3 ± 10.5	1.03 ± 1	14.2 ± 1.8	24.5 ± 5.5	0.6 ± 0.15
Minimum velocity, mean (cm/s)	10.8 ± 7.7	9.6 ± 4.5	1.46 ± 0.99	10 ± 4	10 ± 4.4	1.03 ± 0.25
Diameter, mean (mm)	6.6 ± 0.1.6	6.2 ± 2.2	1.11 ± 0.31	6.8 ± 1.1	6.6 ± 1.5	1.04 ± 0.21
Flow pattern						
Respirophasic and pulsatile	1 (7.7%)	2 (16.7%)		0 (0%)	3 (50%)	
Respirophasic	2 (15.4%)	5 (41.7%)		1 (16.7%)	1 (16.7%)	
Decreased	0 (0%)	1 (8.3%)		3 (50%)	0 (0%)	
Pulsatile	3 (23.1%)	3 (25%)		0 (0%)	1 (16.7%)	
Continuous	7 (53.8%)	1 (8.3%)		2 (33.3%)	1 (16.7%)	

**Table 8:** Outcome measures of the cohort. ECMO: extracorporeal membrane oxygenation, ICU: intensive care unit.

	Overall Population *N* = 19
Bleeding at cannulation site	3 (15.8%)
Compartment syndrome/fasciotomy	
During ECMO	1 (5.3%)
After decannulation	1 (5.3%)
Thrombectomy	
During ECMO	3 (15.8%)
After decannulation	1 (5.3%)
Arterial vessel repair	
During ECMO	1 (5.3%)
After decannulation	1 (5.3%)
ICU mortality	9 (47.4%)

## Data Availability

The data presented in this study are available on request from the corresponding author.
